# Theoretical foundations and mechanisms of health systems responsiveness: a realist synthesis

**DOI:** 10.1016/j.ssmhs.2025.100061

**Published:** 2025-06

**Authors:** Tolib Mirzoev, Ana Manzano, Irene Akua Agyepong, Bui Thi Thu Ha, Linda Lucy Yevoo, Elizabeth Awini, Anthony Danso-Appiah, Leveana Gyimah, Do Thi Hanh Trang, Le Minh Thi, Kimberly Lakin, Sumit Kane

**Affiliations:** a London School of Hygiene and Tropical Medicine, UK; b University of Leeds, UK; c Ghana Health Service, Accra, Ghana; d Hanoi University of Public Health, Viet Nam; e University of Ghana, Legon, Accra, Ghana; f Mental Health Authority, Accra, Ghana; g University of Melbourne, Australia

**Keywords:** Health system, Responsiveness, Theory, Realist synthesis, Ghana, Vietnam

## Abstract

Health systems responsiveness is a key health systems goal, operationalised as an outcome measured across domains such as dignity and confidentiality. It also reflects values and inputs towards improved health. In this realist synthesis, we critically reviewed underpinning theories, examined mechanisms, and propose a theoretical model of health systems responsiveness. Four theories enhance the understanding of responsiveness: Complex Adaptive Systems, Human Agency, Health Equity, Justice and Social Accountability, and Cultural Capital. It is a social construct reflecting what people expect from the system within social and cultural contexts; and what systems actors (providers, managers) expect from people in the context of standards of care and available resources. Responsiveness is shaped by the societal context of care and the health systems context. Domains of responsiveness are inter-related and comprise values, processes and resources. Our proposed theory highlights the importance of favourable social and organisational contexts in triggering sense of agency, literacy and empowerment that contribute to enhanced people’s capacity to engage with health systems and health system’s capacity to respond to people’s expectations. We hope it offers a useful heuristic to inform efforts in improving health systems responsiveness.

## Highlights

• This paper reviews mechanisms and theories of health systems responsiveness.
• Responsiveness is a social construct around people’s agency to ensure accountability.
• Responsiveness domains, inter-relate and include values, processes and resources.
• The context of care and the care context influence health systems responsiveness.
• A middle-range theory of responsiveness can inform future research and practice.

## Introduction

Health systems responsiveness is “…*when institutions… are cognisant and respond appropriately to the universally legitimate expectations of individuals… safeguarding of rights of patients to adequate… care*” (de Silva, 2000) ^p.3^. It is considered a socially constructed phenomenon, essentially reflecting experiences of service users against the non-medical or social aspects of healthcare such as dignity and confidentiality (Mirzoev and Kane, 2017). Improved health systems responsiveness can contribute to improved quality of care, utilisation of available services and performance of the health system as a whole (Busse et al., 2012, Valentine et al., 2003, Valentine and Bonsel, 2016). However, health systems responsiveness is one of the least studied health systems goals (WHO, 2000) and while the literature on its various aspects is growing, it remains limited (Mirzoev and Kane, 2017, Cleary et al., 2013, Berlan and Shiffman, 2012, Lodenstein et al., 2016).

The most widely accepted framework for understanding and measuring responsiveness is the WHO 2000 framework, which comprised different domains (dignity; autonomy; confidentiality; prompt attention; quality of amenities; access to support networks; communication and choice of service provider) (de Silva, 2000, Darby et al., 2000, Letkovicova et al., 2005), with scholars subsequently proposing further domains of trust (Mirzoev and Kane, 2017), effective care (Forouzan et al., 2011), continuity (Bramesfeld et al., 2007) and human rights (Gostin et al., 2003). An original taxonomy by Murray and Frenk categorised these domains into two components: (a) respect for persons (i.e. dignity, confidentiality and autonomy to decide about their own health); and (b) client orientation (including prompt attention, access to social support networks, quality of basic amenities and choice of provider) (Murray and Frenk, 2000). Valentine et al. proposed the distinction between interpersonal domains (dignity, autonomy, communication and confidentiality) and structural domains (quality of basic amenities, choice, access to social support networks and prompt attention) (Valentine et al., 2008). Surveys to measure health systems responsiveness along these domains in multiple countries (Letkovicova et al., 2005, Valentine et al., 2008, Njeru et al., 2009), revealed prompt attention, dignity and communication as the most important domains and access to social support networks as the least important domain (Valentine et al., 2008). Further studies explored applicability of specific domains to different health areas or programmes such as mental health, HIV/AIDS or pain (Forouzan et al., 2011, Njeru et al., 2009, Rottger et al., 2014, Fiorentini et al., 2015) and have shown that responsiveness can be context-sensitive (e.g. expectations of dignity reflect political, democratic and policy climate (Robone et al., 2011)), vary across actors (e.g. power dynamics between patients and providers (Valentine and Bonsel, 2016); Valentine et al., 2009), health facilities (e.g. public/private (Mirzoev and Kane, 2017); Robone et al., 2011; Valentine et al., 2009; Mirzoev et al., 2021a), and nature of health issues (e.g. chronic, acute or terminal conditions (Forouzan et al., 2011); Njeru et al., 2009; Rottger et al., 2014; Fiorentini et al., 2015). A recent review of health systems responsiveness (Khan et al., 2021) suggested three dominant perspectives in defining health systems responsiveness: (a) unidirectional user-service interface, (b) feedback loops between users and health system, echoing the importance of interactions between the people and health systems (Mirzoev and Kane, 2017) and (c) accountability between the public and the health system, reflecting their importance within health systems (Cleary et al., 2013, Lodenstein et al., 2016). Further work covered different aspects of health systems responsiveness such as what constitutes legitimate and context-specific expectations of responsive health systems (Lakin et al., 2024, Lakin and Kane, 2023, Lodenstein et al., 2013) and influences of actors power and positionality on practices of health systems responsiveness (Kagwanja et al., 2022, Kagwanja et al., 2024).

As an intrinsic health systems goal (Murray and Frenk, 2000, Valentine et al., 2009), health systems responsiveness is typically operationalised as an outcome of health systems performance measured across its different domains. However, health systems responsiveness can also be regarded as a set of value propositions within national health systems (for example, dignity and confidentiality) and also inputs towards achievement of an ultimate health systems goal of improved health (for example, reflected in processes of communication and quality of amenities). Thus health systems responsiveness is not only a desired outcome, but it is also an accumulation of continuous processes to adapt and improve practices and structures in response to people’s legitimate expectations. The literature also contains limited examinations of specific mechanisms of how health systems responsiveness actually works in adequately recognising and addressing people’s legitimate expectations (de Silva, 2000). Most empirical work on health systems responsiveness is within the boundaries of individual health programmes (Forouzan et al., 2011, Njeru et al., 2009, Rottger et al., 2014), and little is known about systems level responsiveness at the intersection of more than one programme, for example maternal health and mental health (Mirzoev et al., 2021a, Mirzoev et al., 2021b). Greater clarity on the nature of health systems responsiveness and its domains and mechanisms, can further enhance transferability of responsiveness across different settings and usefully inform efforts to improve health systems responsiveness.

In this paper, we attempt to bridge these knowledge gaps by advancing the understanding of the nature of health systems responsiveness and specific mechanisms of health systems responsiveness, drawing on available literature and empirical insights from research on health systems responsiveness to maternal mental health in Ghana and Vietnam. Thus, the objectives of the paper are to critically review the concept and underpinning theoretical foundations of health systems responsiveness, perform a ‘deep dive’ into the mechanisms through which the health systems responsiveness operates and based on the above, propose a theoretical model of health systems responsiveness.

## Methods

We report results from a realist synthesis (Mirzoev et al., 2021b) which was embedded within a wider collaborative realist evaluation of the health systems responsiveness to mental health needs of pregnant women in Ghana and Vietnam (Mirzoev et al., 2021a). Realist synthesis is an evidence review approach which follows the realist logic of building programme theories to explain which contexts trigger specific mechanisms in certain contexts and how these interactions may produce intended or unintended outcomes (Rycroft-Malone et al., 2012, Pawson et al., 2004, Pawson et al., 2005). Similar to realist evaluations, realist syntheses follow the realist philosophy which is underpinned by a generative understanding of causality and rely on human volition to understand how programmes, policies or interventions work (Greenhalgh et al., 2017a). However, while realist evaluations primarily use primary data, realist syntheses use primarily secondary data from published and unpublished literature (Greenhalgh et al., 2017a). In contrast with traditional systematic reviews, realist syntheses are *“…not a method or formula, but a logic of enquiry that is inherently pluralist and flexible, embracing both qualitative and quantitative, formative and summative, prospective and retrospective…” (*Pawson et al., 2005*)* ^p.32^. One advantage of a realist approach to evidence synthesis is its focus on unpacking how interventions or programmes work while allowing for flexibility in searching, screening and data extraction as compared with traditional systematic reviews. Furthermore, realist synthesess are inclusive of the broader types of studies including grey literature (Rycroft-Malone et al., 2012, Pawson et al., 2004, Pawson et al., 2005). Specific steps recommended in realist syntheses are similar to those of the systematic reviews: identifying review questions, searching for primary studies, quality assessment, data extraction, synthesising results and dissemination (Rycroft-Malone et al., 2012, Pawson et al., 2004). However, these steps are not linear and the process is highly iterative, allowing for the necessary customization needed in specific complex research areas, for example moving away from methodological appraisal of quality of evidence towards degree of richness of description of a specific phenomenon (such as research partnerships (Jagosh et al., 2014)) and whether substantive theory should drive the development of review protocols from the outset or should follow data immersion (Jagosh et al., 2014). Further distinctive features of the realist syntheses are: wide variation in the degrees of stakeholder engagements, comprising informal consultations in formulating initial theories and developing policy recommendations (Pawson et al., 2005) and more formal data collection and analysis for example using in-depth interviews and surveys, to inform theory refinement (Dunn et al., 2021) and even theory testing (Cooper et al., 2017).

In the realist synthesis, we sought to answer two inter-related questions: (a) Which substantive theories underpin the understanding of health systems responsiveness in the literature, and how do they inform these interpretations? and (b) In what way does responsiveness work for different health systems actors (service users, providers and managers) in the contexts of LMICs? To answer these questions, initially, we planned to conduct the realist synthesis during the first year, but given paucity of published knowledge about responsiveness; and in recognition of importance of emerging empirical insights, early on we realised that the realist synthesis will have to run alongside the wider realist evaluation study which included phases of initial analyses, co-production of interventions to improve health systems responsiveness and pilot-testing of these interventions. Wong et al. (2024) explained that practicalities (e.g., time, staffing, available evidence) can interfere with intended sequencing when a realist synthesis and realist evaluation are combined. If it is not possible to sufficiently develop the programme theory from the realist synthesis before embarking on primary data collection, flexibility and judgment must be applied on final sequencing (Wong et al., 2024).

We were guided by the RAMESES publications standards for realist syntheses (Wong et al., 2013) and followed a four-step process similar to Cooper et al (Cooper et al., 2017). Step 1 involved mapping of theoretical underpinnings of health systems responsiveness (Rycroft-Malone et al., 2012, Cooper et al., 2017), through initial screening of literature. During step 2, we formulated initial theories of how responsiveness works, drawing on current theorisation of responsiveness in the literature, team discussions and consultations with key health systems actors (Pawson et al., 2005, Cooper et al., 2017) in Ghana and Vietnam. In step 3, we refined programme theories through iterative engagements with the literature alongside engagements with key actors from Ghana and Vietnam. Our engagements with key actors utilised combinations of surveys to assess health systems responsiveness in each context using an adaptation of the WHO questionnaire which included an additional domain of trust, reflecting our theorisation of responsiveness (Mirzoev and Kane, 2017, Mirzoev et al., 2021a, Vui et al., 2024); in-depth interviews with purposefully-identified health systems actors (service users, providers and decision-makers); and intervention co-production workshops with key actors in each country. Finally, step 4 entailed testing of programme theories using insights from evaluations of implemented interventions in Ghana and Vietnam.

### Inclusion/exclusion criteria and searches

Our engagement with the literature was iterative, phased, and incremental. Global Health, Ovid MEDLINE(R) and Google Scholar databases were searched. There were no restrictions on populations, geography or study type and grey literature was included. Neither were there restrictions on language, with the intention of including studies if they were in the team’s languages (English, Spanish, French, Russian and Vietnamese). We excluded studies published before 2000 i.e. when the WHO concept of responsiveness was created.

**Fig. 1:**
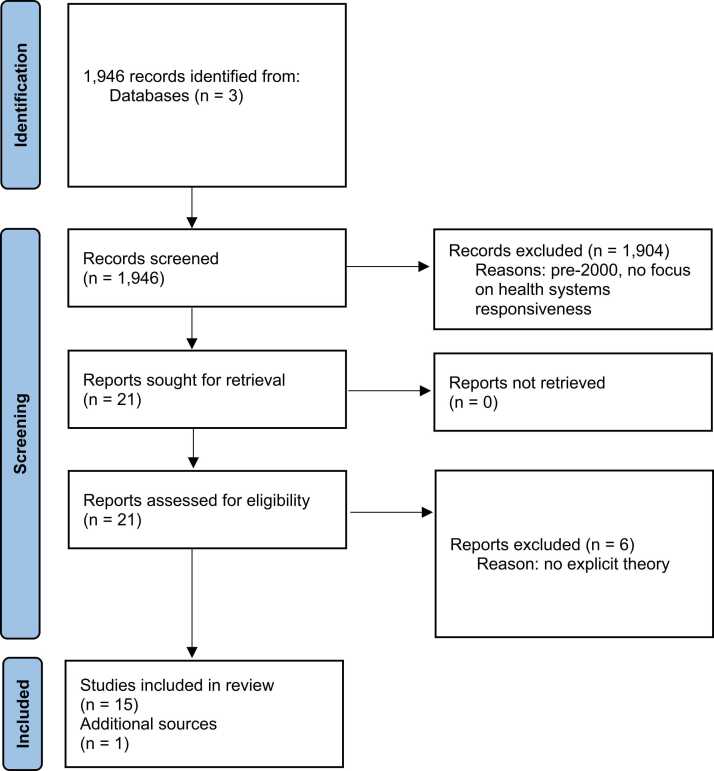
PRISMA flow diagram.

We started with a rapid review of knowledge which aimed to identify the underlying theoretical bases of health systems responsiveness to inform deliberations by researchers. Initial searches in September 2020 were guided by broad search parameters (Box 1) and initial title and abstract screening and subsequent full-text screening yielded a selection of 15/1946 papers with one additional publication included after following up on citations. As shown in the PRISMA flowchart (Fig. 1), the primary reason for exclusion at this point of the review was the absence of explicitly articulated theoretical foundations of health systems responsiveness.

Box 1 **search keywords.**

**Table:** 

conceptual* , theor* , framework, model* or analy* or evaluat* , theory, health* , system* , respons* confiden* , satisf* , prompt, timely, lengthy, delay* , dignity, choice, decision, decide, access, quality, access, accountab* , health service responsiveness, quality of health care, personal autonomy, respect, health services accessibility, *respect, *trust, *confidentiality, delivery of health care, healthcare responsivity

This was followed by more in-depth iterative searches related to different domains of health systems responsiveness (such as dignity, confidentiality), as well as on two broad aspects of health systems responsiveness corresponding to our theoretical framing (internal and external interactions) (Mirzoev and Kane, 2017, Mirzoev et al., 2021b). This knowledge informed gleaning of initial programme theories, and primary data collection and analysis for the development and refinement of the programme theories.

The subsequent stages of the realist synthesis over the course of the study (2020–2024) were guided by different iterations of programme theories. For example, one of subsequent searches of the Global Health, Ovid MEDLINE(R) and Mental Health Innovation Network databases conducted in February 2022 for evidence of integration, joint working, communication guidelines and policy interventions on maternal mental health of relevance to Sub-Saharan Africa and SE Asia yielded 44 results. Further specific theories, for example around integration of maternal mental health and stigma surrounding mental health during pregnancy and postpartum gleaned in the initial reviews were modified with support from the empirical data across countries.

Various aspects of emerging programme theories and themes from the empirical data also became distinct parallel and nested ‘projects’, which indirectly informed the relationship between realist synthesis and wider realist evaluation. These included: review of the burden of maternal mental health in sub-Saharan Africa (Awini et al., 2023), a systematic review of screening tools for mental health during pregnancy and postpartum in sub-Saharan Africa (Gyimah et al., 2024) and South Asia followed by validation of one screening tool (Self-Reporting Questionnaire, SRQ-20) (Do et al., 2023).

### Data extraction, analysis and synthesis

Due to the iterative, large and ever evolving nature of our review, we did not consistently apply one structured data extraction format or template throughout the review. Instead, data was extracted following individual domains of responsiveness and then subsequently following individual components of the emerging programme theories. Relevant fragments of the data (such as empirical evidence or experiences supporting parts of context-mechanism-outcome configurations) were continuously extracted as we progressed through different iterations, and at different points the analysed data was consolidated in an interim report or brief to inform the next steps.

Our approach to data analysis started with a more inductive engagements with the literature and thematic analysis of empirical data from initial data collection and analysis to identify initial programme theories. Subsequently, this evolved into a retroductive approach as a more traditional approach to realist analyses (Wong et al., 2013, Greenhalgh et al., 2017a) with elements of abduction of relevant pieces of theory from the literature and the empirical data. We synthesised data against the emerging initial programme theories, which aimed to cover relevant aspects of responsiveness to the context of Ghana and Vietnam against our understanding of internal (within health systems) and external (people-health systems) interactions (Mirzoev and Kane, 2017, Mirzoev et al., 2021a, Mirzoev et al., 2021b) as two key elements of health systems responsiveness. The research teams in Ghana and Vietnam worked independently, regularly converging to ensure mutual sharing and learning. Supplementary File 1 provides an example of an early round of visualisation of programme theories which were emerging from different sources in June 2021. These contain inductively-identified themes, which were collated against broader themes, and which over time evolved into distinct programme theories such as stigma around maternal mental health (Thi et al., 2024) and integration of maternal mental healthcare in Vietnam (Trang et al., 2024a). Over time, the emerging theoretical and empirical insights were consolidated into a middle-range theory of health systems responsiveness reported later in this paper.

### Ethics

The study was approved by the Institutional Review Boards of the Hanoi University of Public Health (Decision Number 33/2022-YTCC-HD3), Ghana Health Service (ref GHS-ERC 012/03/20), London School of Hygiene and Tropical Medicine (ref 22981), and the University of Leeds (ref MREC 19–051). Written informed consent was obtained from all study participants before fieldwork, and their anonymity and confidentiality were preserved during data analysis and in reporting findings.

## Results

The majority of literature on responsiveness is empirical and predominantly focuses on either measuring degree of health systems responsiveness against the different domains (Letkovicova et al., 2005, Valentine et al., 2008, Njeru et al., 2009) or different propositions for additional domains of responsiveness (Forouzan et al., 2011, Njeru et al., 2009, Rottger et al., 2014, Fiorentini et al., 2015). Recent theoretical literature focused on proposing further conceptual framing (Mirzoev and Kane, 2017, Gostin et al., 2003), synthesising knowledge on health systems responsiveness (Mirzoev and Kane, 2017, Khan et al., 2021), advancing specific aspects of health systems responsiveness such as legitimate expectations (Lakin et al., 2024, Lakin and Kane, 2023, Lakin and Kane, 2022) and improving responsiveness at the intersection of different health programmes (Mirzoev et al., 2021a). However, theoretical and conceptual literature covering health systems responsiveness is limited, with only few recent attempts to advance this concept. This may reflect the complex nature of responsiveness as compared with more tangible health systems goals of achieving better health outcomes, efficiency or fair financial contribution (WHO, 2007).

### Conceptualising health systems responsiveness

Health systems responsiveness is one of the key fundamental health systems goals (Murray and Frenk, 2000, WHO, 2007), operationalised as an outcome of health systems performance. Originating from the literature on quality of care and patient satisfaction (Valentine et al., 2003), health systems responsiveness is often conceptualised as a legitimate outcome of a healthcare process (Robone et al., 2011, WHO, 2007, WHO, 2010), measured through people’s reflections on their experiences of care encounter (Mirzoev and Kane, 2017, Robone et al., 2011, Valentine et al., 2009) echoing that “…consumer satisfaction …in the assessment of health services reflects the responsiveness of health systems” (WHO, 2010) ^p.90^ (p.90). However, health systems responsiveness also encompasses the process of ensuring that healthcare services are rendered with due attention to its non-clinical aspects such as dignity and confidentiality (Forouzan et al., 2011, Valentine et al., 2008). For example, collecting, analysing and responding to patient feedback is a process that can encourage institutional change by adjusting procedures; as continuous education and quality assurance processes can enhance health workers’ ability to provide respectful care. This duality infers a complex and dynamic nature of responsiveness: it is not only a desired outcome, but it is also an accumulation of continuous intrinsic processes to adapt and improve practices and structures in response to patient needs and expectations.

Health systems responsiveness should be considered a socially-constructed phenomenon (Mirzoev and Kane, 2017), which is built on the premise of people having sufficient agency (and associated literacy) to ensure and demand responsiveness as reflected in different domains. This is because the construct of responsiveness is shaped by social, cultural, and contextual factors, which determine and shape people’s experiences of care they receive against their initial expectations. For instance, the perception of what constitutes respectful behaviour in health systems is influenced by cultural norms, societal expectations, and individual experiences (Lakin et al., 2024, Lakin and Kane, 2022). While in some cultures, it is respectful for healthcare providers to address patients by their titles and surname, in others, using first names is more a more established convention to ensure approachability while also maintaining respect.

Responsiveness operates within the context of initial expectations of what people expect from the health facilities/systems as a benchmark for people’s assessments of health systems responsiveness. However, it also operates in the context of what health systems actors (service providers, managers) expect from people (Mirzoev and Kane, 2017). For example, patients expect health systems to treat them according to their understanding of respect and dignity, while also benefiting from empathy and care during health service provision. On the other hand, service providers typically expect patients to follow their advice and adhere to their recommendations. Meanwhile, health managers are often focused on maximizing the value of available resources in achieving best possible performance and outcomes. Thus, people’s expectations typically reflect their cultural, relational, spatial and time-bound perspectives within the social groups they live in (Lakin and Kane, 2023), which are also embedded within the structural health systems contexts (i.e. norms and standards of care, resources, staff skills, amenities) including perceptions of degree of people’s legitimate mandate from health service providers (Lodenstein et al., 2016).

Practices of service providers are also shaped by their values and attitudes towards different population groups (Schulze, 2007) as was shown in a study which reported biased perceptions and negative attitudes of service providers towards vulnerable and marginalised street children and youth in Kenya (Lonnie et al., 2021). This exemplifies that health systems responsiveness as a process and an outcome can be driven by responsiveness as value.

From the perspective of maternal mental health, our empirical data highlights two condition-specific contexts which shape responsiveness as a desired outcome for pregnant women with mental health conditions:

(a) The societal context or *the context of care.* The societal or structural stigma surrounding the mental health significantly influences health systems responsiveness (Thi et al., 2024). Stigmatising attitudes towards people with mental conditions are pervasive within local communities and affect access to and quality of care. We also found widespread stigma towards health professionals. In Vietnam psychiatrists are often referred to as “*bac si tam than”*, which translates to “*doctors who treat madness”* and in Ghana the community psychiatric nurses reported that other colleagues call them “*the crazy people nurses*”*.* These derogatory terms highlight how the context of stigmatized care can constrain provision and utilization of health services.
(b) The health systems context or the *care context*. The degree of integration between maternal and mental health services and programmes (Trang et al., 2024b) plays a major role in shaping health systems responsiveness to maternal mental health needs. Integrated care for maternal mental health is often lacking due to gaps in policy environment, insufficient staff expertise and limited resources available. For example, in Vietnam and Ghana - as in many countries - maternal health services operate independently from mental health services, and these services are often treated as separate issues rather than interconnected aspects. The availability of trained staff who can address both maternal and mental health needs is limited and efforts to integrate these services are hampered by resource constraints, resulting in undesired clinical outcomes and lower health systems responsiveness.

### Conjunctive theoretical foundations of health systems responsiveness

The concept of health systems responsiveness is informed by several substantive theories that help to provide a comprehensive understanding of the concept, integrating aspects from health systems, organisational studies and sociology. As Tsoukas (2017) argued, there are concepts that cannot be dealt with by simplifying theory but instead “conjunctive theorizing” is necessary for a framework to embrace complexity instead of unnecessary simplification (Tsoukas, 2017). For this reason, in the case of responsiveness, four distinct but interrelated theoretical perspectives - Complex Adaptive Systems (CAS) (Plsek and Greenhalgh, 2001), Human Agency (Emirbayer and Mische, 1998), Health Equity, Justice and Social Accountability (Lodenstein et al., 2016, Lodenstein et al., 2013, Abimbola et al., 2022), and Cultural Capital (Bourdieu, 1986) - can be jointly applied to enhance the understanding of theoretical foundations of responsiveness. Due to the complexity and multi-faceted nature of health system responsiveness, the theoretical framework became clearer not during the earlier iterations of searches and analyses as initially planned, but later in the process, as primary data collection analysis progressed and our programme theories and consolidated middle-range theory matured.

A complex adaptive systems theory can provide an overall framework to help understand health systems as comprising a collection of individual agents with freedom to act within flexible and unstable boundaries, often acting in unexpected ways (Plsek and Greenhalgh, 2001, Paina and Peters, 2012, Plsek and Wilson, 2001, Agyepong et al., 2012). Since their actions are interconnected, one agent’s actions change the context for other agents (Plsek and Greenhalgh, 2001, Paina and Peters, 2012). From the perspective of health systems responsiveness, the nature, contents and length of interactions, which are often determined by the service providers (such as in-person or over phone, duration of the discussion and integrated approach for example in addressing maternal mental health needs rather than each issue in isolation) can shape people’s reflections on whether their needs and expectations have been adequately addressed and whether specific attributes of health systems responsiveness – such as dignity and respect – have been maintained in the process of those interactions. Understanding responsiveness through the lenses of interconnected and dynamically interacting components, continuous adaptability and emergence of further patterns of systems behaviour, recognises the complexity of healthcare delivery and interactions between the people and their health systems.

People’s decisions whether to seek healthcare, and subsequent interactions with health systems, are determined by their perceived need for healthcare as well as people’s sense of agency to achieve the desired outcomes. Human agency is a construct which entails a process of social engagement embedded in time and thus informed by ‘*its past (in its habitual aspect), oriented towards the present (as a capacity to conceptualize past habits and future projects within the contingencies of the moment) and oriented towards the future (as a capacity to imagine alterative possibilities)*Emirbayer and Mische, (1998) ^*p.970*^. From the perspective of responsiveness, habitual aspects of agency inform people’s initial expectations which also serve as drivers for engaging with health systems and benchmarks for self-reflections on the degree of health systems responsiveness. This theory emphasizes the importance of empowering patients and healthcare providers to actively participate in decision-making to ensure that preferences and values for both actors are respected. For example, effective communication between patients and providers are central to the concept of responsiveness. Consequently, implementing patient advisory structures and consultations - such as patient councils, boards, forums and committees - is likely to strengthen these interactions as part of improving responsiveness. Of course, all these require appropriate socio-political, institutional and bureaucratic environments which can facilitate patient and provider empowerment.

Health Equity and Justice theories (Benfer, 2015, Wiley, 2016) are integral to health systems responsiveness because they help understanding and addressing the diverse needs of the populations they care for, including vulnerable and/or marginalized populations (e.g., migrants, low-income individuals, women and children, patients with stigmatizing diseases such as infectious and mental health illness). Collectively, with social accountability theory, they aim to create fair, inclusive, and responsive health systems. Social accountability emerged as a prominent theoretical perspective in the literature on responsiveness (Khan et al., 2021), though it was not always explicitly mentioned. People can use different strategies to hold health systems to account against their expectations, for example dialogue and advocacy, information collection and analysis, presentations to officials and service providers, action planning or negotiation and follow up if there was no adequate outcome (Lodenstein et al., 2016, Lodenstein et al., 2013). People’s actions to demand and uphold principles of accountability are intrinsically linked to perceptions of how legitimate citizens perceive themselves within their contexts (Lakin and Kane, 2023, Lakin and Kane, 2022) and are inevitably affected by the way providers perceive and value a formal mandate (Lodenstein et al., 2016, Lodenstein et al., 2013) within the organizational context of health facilities which determines intra-system bureaucratic accountability (Cleary et al., 2013), which ultimately affects health systems responsiveness. This theoretical perspective highlights the importance of transparency, participation and accountability to diverse community needs and expectations. For instance, programmes promoting the incorporation of user feedback into health service design and planning are likely to support the long-term desired outcome of increasing responsiveness because they ensure that services are continuously aligned with people’s needs and preferences. This engagement also fosters trust between the community and service providers, which is a key mechanism of health systems responsiveness (Mirzoev and Kane, 2017). However, in highly fragmented health systems, where services are provided by multiple, uncoordinated programmes across public and/or private institutions, collecting and comprehensively responding to feedback can be challenging. Effective user feedback systems require effective infrastructures for data management, which are often lacking in LMICs. Furthermore, cultural barriers and fear of repercussions may discourage patients from sharing their voices (Huque et al., 2021, Mirzoev et al., 2021c) and service providers may often resist feedback, if they perceive it as threat to themselves or their institutions (Ha et al., 2015).

Finally, Bourdieu's theory of cultural capital posits that individuals possess cultural resources (such as education, knowledge, and cultural tastes) that can be converted into social advantage and subsequent action (Bourdieu, 1986). In relation to health systems responsiveness, this can translate into ability and willingness to engage with health systems, for example as part of care seeking. Within health systems, cultural capital can manifest in different forms, including embodied state (e.g., linguistic skills, knowledge, cognitive abilities) as assets that can enhance people’s ability to process medical information, navigate health systems, and effectively communicate with service providers, objectified state (e.g., access to information and resources like books, leaflets, digital information), and institutionalized state (e.g., health workers’ educational credentials and professional training) (Bourdieu, 1986). This theory underscores the impact of cultural factors on health behaviours and interactions. Understanding the role of cultural capital highlights the importance of literacy, and agency in improving health systems responsiveness within socio-economic, and institutional culture contexts.

From the perspective of maternal mental health, our empirical findings also highlight the importance of literacy, agency and empowerment of both pregnant and postpartum women as well as health workers, in ensuring health systems responsiveness. In Vietnam, women often were not recognizing symptoms of common mental health conditions during pregnancy as requiring attention. Due to limited mental health literacy, these symptoms are often normalised as part of the hormonal alterations of pregnancy and post-partum. In Ghana, mental health symptoms in pregnancy were often attributed to supranatural causes. These explanations are commonly linked to undesired female social behaviours (e.g., evil eye if women do not hide pregnancy and behave modestly). Individuals from lower socio-economic backgrounds face additional challenges as they have limited access to educational opportunities, and health-related resources, especially resources about conditions stigmatised in some cultures such as mental health. This stigma prevents women from seeking help for maternal mental health problems, in a healthcare context where patriarchal norms are likely to limit women's autonomy in making healthcare decisions. Additionally, in low resource settings, healthcare staff professional credentials specialised in neglected aspects of health systems such as mental health are scarce.

### Mechanisms of health system responsiveness

The ‘achievement’ of health systems responsiveness to people’s legitimate expectations is typically measured through people’s self-reflections along the different domains. Each domain can be regarded as a mechanism in the realist sense, which are defined as combination of reasoning and resources (Dalkin et al., 2015, Pawson and Tilley, 1997), thus also bridging an earlier-introduced taxonomy of respect for persons (including dignity, confidentiality and autonomy to decide about their own health) and client orientation (including prompt attention, access to social support networks, quality of basic amenities and choice of provider) (Murray and Frenk, 2000), which were subsequently termed as interpersonal and structural domains, respectively (Valentine et al., 2008). However, the distinction between reasoning and resources of domains of responsiveness is worth disentangling further, because each domain also encompasses multiple associated resources and further underlying causal chains of reasoning and some may even represent contextual drivers of health systems responsiveness:

• *Dignity* is about respect in being treated in a non-stigmatizing manner and as an equal human being, appropriate tone and taking patients seriously, privacy and maintaining individuality (Valentine et al., 2003, Forouzan et al., 2011).
• *Autonomy* includes the right to self-determination, freedom to choose, informed consent and right to refuse treatment (Valentine et al., 2003), participation in the care process and a feeling of equal power (Forouzan et al., 2011).
• *Prompt attention* is about being treated as soon as necessary, which besides adequate timing, involves a close relationship, insightful listening, empathy within a trusting relationship and thoughtful care (Forouzan et al., 2011).
• *Quality of amenities* includes the (contextual) environment provided by the heath service such as warmth, hygiene, facilities, aesthetics and comfort (Valentine et al., 2003) with the latter also being a possible mechanism in some circumstances if it promotes effective communication, satisfaction and possibly contribute to enhanced trust.
• *Confidentiality* is about protecting personal information while ensuring that all treatment is in an understandable language (Valentine et al., 2003).
• *Access to support networks* is about social, physical and spiritual support and advocacy from family and community and relevant organizations.
• *Choice of service provider* includes an option to get another opinion, although this can be challenging for example due to limited resources, geographical distance, referrals, insurance or litigation (Valentine et al., 2003).
• *Trust* denotes the degree of perceived confidence from the people that the health service providers and the wider health system are likely to perform according to their expectations with regards to clinical and social aspects of healthcare (Mirzoev and Kane, 2017, Ezumah et al., 2022, Gilson, 2003, Gilson et al., 2005, Hurley, 2006)

Our empirical data on health systems responsiveness to maternal mental health needs highlighted a generally higher level of perceived health systems responsiveness at primary healthcare level in Ghana as compared with Vietnam, with overall mean scores of all domains being 64.85 out of 80 (81 %) and 2.86 out of 4 (71.5 %), respectively. In Ghana, the most highly scored individual domains of responsiveness were dignity and trust, whereas the least scoring domain was choice of service provider, and people with mental health conditions were significantly likely to score lower. The importance of dignity and trust may reflect a high degree of empathy from health workers and stronger social bond amongst healthcare staff and pregnant women within communities and primary healthcare facilities. In Vietnam, the most highly scored elements of responsiveness were social support, trust and dignity, whereas the lowest rated were choice of provider, prompt attention and communication (Vui et al., 2024). These findings reflect the nature of Vietnam’s health system, characterised by centralised governance and decision-making, embedded within strong cultural and religious value-driven society. They may also suggest a possible hierarchy of the different elements of health systems responsiveness at the primary healthcare level in Vietnam. Our empirical insights thus highlight that perceptions of the different domains of health systems responsiveness, and possibly degree of their perceived importance, can vary across social and health system contexts and for different population groups (Lakin et al., 2024).

Some of these domains are clearly interrelated and are mutually reinforcing. For instance, prompt attention can foster a sense of dignity and trustworthy relationships; confidentiality can determine the degree of trust to specific service providers, shaping their reputation and credibility; and choice of service provider is likely to be driven by quality of amenities and access to social support networks. It is, therefore, important to disentangle these relationships and identify which might be the ‘core’ mechanisms through which health systems responsiveness can be achieved for specific groups within different settings and in condition-specific contexts of care.

Building on earlier classifications of domains of health systems responsiveness as comprising respect for persons and client orientation (Murray and Frenk, 2000) and interpersonal and structural domains (Valentine et al., 2008), we propose that domains of health systems responsiveness can be categorised into three broad groups:

1. *Underlying values* such as dignity, autonomy, attention. From the realist perspective, these effectively constitute potential reasoning mechanisms that are triggered following people’s experiences of care
2. *Potential processes* such as communication. From the realist perspective, these trigger the sense of attention and assurance that people’s voices are heard, and their needs are sufficiently recognised and addressed
3. *Available resources* such as access to networks, quality of amenities and choice of providers. From the realist perspective, these are best understood as combinations of contextual (health systems and societal) organisation and structures.

The achievement of health systems responsiveness is also fundamentally about capacity of: (a) people to sufficiently engage with their health systems and (b) the health system, via frontline health workers and health managers, to recognise and address people’s expectations. The health systems literature highlights two underlying questions around capacity (capacity of whom and capacity in relation to what tasks) as well as the importance of intersecting individual and collective-level capacities (Mirzoev et al., 2022, Green and Bennett, 2007, UNDP, 2008). While individual capacities are more easily understood as ability to perform certain tasks, the notion of collective capacity in the context of health systems responsiveness recognises that people are embedded within their families and communities, and service providers are embedded within contexts of health facilities and wider systems. All these actors are further situated within the condition-specific context of care and administration of the care context, shaping how responsiveness is enacted and experienced.

Consequently, theoretical underpinnings of capacity and value judgements related to responsiveness are likely to differ between the ‘people’s side’ and the ‘health system’s side’ of health systems responsiveness. Issues of agency, literacy and empowerment can determine people’s capacity to engage with their health systems and hold their systems to account. On the other hand, issues of commitment, empathy, care, effective communication and lack of prejudices alongside technical clinical expertise can be particularly relevant on the ‘health systems’ side to determine capacity (and willingness) of health workers to recognise and address people’s expectations.

## Discussion

In this paper, we have reviewed knowledge around health systems responsiveness, aiming to clarify the underlying theoretical bases of health systems responsiveness and advance the understanding of how health systems responsiveness works to produce its intended outcomes for different health systems actors (service users, providers and managers) in the contexts of LMIC. A widely accepted definition of health systems responsiveness is “…*when institutions… are cognisant and respond appropriately to the universally legitimate expectations of individuals… safeguarding of rights of patients to adequate… care*” (de Silva, 2000) ^p.3^. Being a least studied health systems goal, health systems responsiveness is about people’s non-medical or social aspects of healthcare (de Silva, 2000, Mirzoev and Kane, 2017, WHO, 2000, Darby et al., 2000) The literature on responsiveness is growing though still limited and is particularly scarce on theoretical foundations of responsiveness and explanatory studies of how responsiveness actually works are limited. Four distinct but interrelated theoretical perspectives - Complex Adaptive Systems (CAS) (Plsek and Greenhalgh, 2001), Human Agency (Emirbayer and Mische, 1998), Health Equity, Justice and Social Accountability (Lodenstein et al., 2016, Lodenstein et al., 2013, Abimbola et al., 2022), and Cultural Capital (Bourdieu, 1986) – have been conjointly applied to enhance the understanding of health systems responsiveness.

The domains of health systems responsiveness are inter-related and mutually-reinforcing realist mechanisms of health systems responsiveness. An earlier taxonomy suggested the distinctions between interpersonal or respect for persons and structural or client orientation domains (Murray and Frenk, 2000, Valentine et al., 2008), and from a realist perspective we propose that domains of health systems responsiveness comprise three broad groups: (a) *underlying values* e.g. dignity, autonomy, attention; these constitute potential reasoning or mechanisms that are triggered following people’s experiences of care; (b) *potential processes* e.g. communication; these trigger the sense of attention and assurance that people’s voices are heard, and their needs are sufficiently recognised and addressed and (c) *available resources* e.g. access to networks, quality of amenities and choice of providers; these are best understood as combinations of contextual (health systems and societal) organisation and structures.

We also argue that achievement of intended outcomes of health systems responsiveness is fundamentally about capacity of: (a) people to sufficiently engage with their health systems and (b) of the health systems, via frontline health workers, to recognise and address people’s expectations.

A key output from this realist synthesis is a proposed middle-range theory of health systems responsiveness which is reported next.

### Middle-range theory of health systems responsiveness

**Fig. 2:**
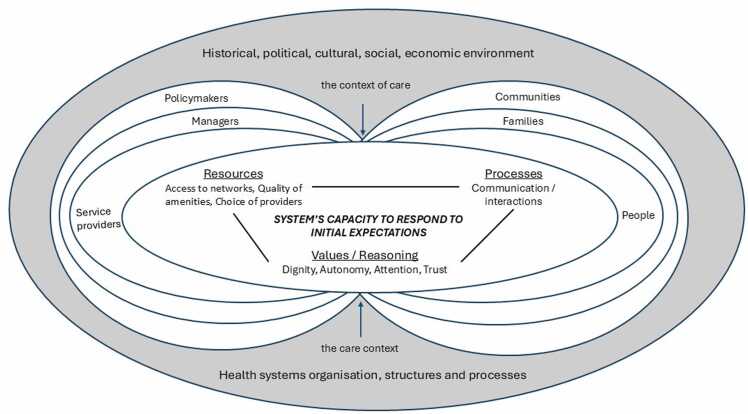
Middle-range theory of health systems responsiveness (adapted from (Mirzoev and Kane, 2017)).

Drawing on the literature and empirical insights from Ghana and Vietnam, we propose the following middle-range theory of how health systems responsiveness works to produce its intended outcomes in its respective contexts (see Fig. 2):

*In context of shared societal and health systems values of dignity, autonomy and respect, alongside related prioritisation of equity, justice and human rights; and adequately resourced, supported and committed health workers who recognise and aim to address people’s expectations of responsive health systems as legitimate, and within the condition-specific societal and health systems contexts of care…; continuous and effective interactions between the people (clients) and their health systems via frontline health workers, tailored to the unique demands of specific conditions…; can trigger a sense of enhanced agency, improved literacy and empowerment from the people and a sense of empathy and commitment to improving client care experiences from the service providers…; which will contribute to improved capacity from the people to effectively engage with their health systems and demand accountability, and improved health system’s capacity to adequately respond to people’s non-medical expectations of care in their particular condition-specific contexts, thus facilitating the achievement of health systems responsiveness as an outcome.*

A central element of realist programme theories are the mechanisms, which comprise resources and reasoning (Dalkin et al., 2015). Implicit mechanisms of health systems responsiveness from a widely used WHO framework comprise different manifestations of the domains of health systems responsiveness. We recognise these, though also acknowledge their different adaptations to specific health areas such as mental health and HIV (Forouzan et al., 2011, Njeru et al., 2009, Rottger et al., 2014, Fiorentini et al., 2015) as well as their context-sensitive nature (Mirzoev and Kane, 2017, Valentine and Bonsel, 2016, Robone et al., 2011, Valentine et al., 2009, Mirzoev et al., 2021a). These domains combine interpersonal and structural issues (Valentine et al., 2008), and arguably comprise *underlying values* (e.g. dignity, autonomy, attention), *potential processes* (e.g. communication) and *available resources* (e.g. access to networks, quality of amenities and choice of providers).

In our programme theory, we highlight key higher-level and more ‘reasoning-type’ mechanisms of enhanced agency (Emirbayer and Mische, 1998), literacy (NHS England, 2017) and related empowerment of people to engage with their health systems, reflecting theoretical perspectives of health systems responsiveness and our empirical insights from Ghana and Vietnam. As set out earlier, human agency can determine people’s initial expectations of healthcare and decisions whether and how to seek healthcare from their health systems. We interpret literacy in a broad sense as “…*the personal characteristics and social resources needed for individuals and communities to access, understand, appraise and use information and services to make decisions about health” (*NHS England, 2017*)* ^*p.4*^*.* Both these can be mutually reinforcing and can aggregate into and through empowerment of pregnant women to seek healthcare for their common mental health conditions, in the context of commitment of health workers to empathetically engage with clients and improve client experiences. We theorise that agency, literacy and empowerment are interrelated and collectively shape capacity or ability of the people (to engage with health systems) and capacity of the health system (to adequately respond to people’s social expectations of care) to ensure achievement of health systems responsiveness.

An overall outcome of health systems responsiveness is the ability or capacity of the health system to recognise and effectively address people’s legitimate expectations of non-medical aspects of healthcare (de Silva, 2000, Robone et al., 2011, Valentine et al., 2009). Different domains of responsiveness (and their subsequent adaptations), apart from potentially representing mechanisms of responsiveness also have been used as detailed measures of responsiveness outcomes (Valentine et al., 2009). Some of the recent work proposed improved internal and external interactions as broader outcomes of health systems responsiveness (Mirzoev and Kane, 2017, Mirzoev et al., 2021a). These interactions are beyond the level of specific domains and may represent more proximal outcomes of responsiveness i.e. preceding the domains. Improved interactions are arguably the outcomes of the exercise of enhanced agency and literacy by the empowered and capable individuals. The distal outcomes of responsiveness would still remain to be improved domains of responsiveness, ultimately contributing towards improved systems accountability, improved healthcare quality, improved utilisation of health services and improved health outcomes (Busse et al., 2012, Valentine et al., 2003, Valentine et al., 2009, Forouzan et al., 2011, Robone et al., 2011, Lodenstein et al., 2013).

The importance of context in triggering the mechanisms has been explicitly emphasised in the realist literature (Pawson and Tilley, 1997, Greenhalgh and Manzano, 2022, Pawson and Manzano-Santaella, 2012, Greenhalgh et al., 2017b). The literature on health systems responsiveness highlights the importance of resource environment; health systems organisation and processes of service delivery including diagnostic and treatment arrangements; wider institutional factors such as welfare provision, democracy and corruption; and characteristics of the population, social and cultural norms (Robone et al., 2011, Valentine et al., 2009), all contributing towards the individual’s agency (Emirbayer and Mische, 1998) and cultural capital (Bourdieu, 1986) as we also found in our empirical data. The societal context also determines whether people’s expectations of care are viewed as legitimate or not within a particular location, at particular time and by specific healthcare workers (Lakin and Kane, 2023, Lakin and Kane, 2022, Lodenstein et al., 2013), often requiring active citizen contestations at the intersection of social, temporal and spatial locations to establish legitimate expectations of care as we found in Vietnam (Lakin et al., 2024). Finally, underlying societal values of dignity, autonomy, prompt attention and related values of equity and human rights can provide underlying bases for implementing and ensuring health systems responsiveness.

Our middle range theory can offer a useful heuristic to advance the understanding of, and structure efforts in improving, health systems responsiveness by focusing on fewer number of core mechanisms. Additionally, policies and practices aimed at improving health systems responsiveness should deploy a systemic approach rather than being constrained by the boundaries of individual programmes. Capacity strengthening efforts in improving health systems responsiveness should focus on both the people and the systems sides of responsiveness to enhance relevant capabilities. It is important, however, to recognise the applicability of different domains of responsiveness to different health areas and conditions such as mental health or adolescent health.

Future research on health systems responsiveness needs to interpret results of health systems responsiveness surveys beyond mechanistic interpretation of responsiveness scores and ranking, and recognise means-ends relationships between the different domains. We also call for future testing of the programme theory on further systems and combinations of programmes to contribute to improved theorisation and operationalisation of the concept. Future work can usefully develop further competencies to ensure health systems responsiveness across different contexts and settings.

### Study limitations and strengths

Given our complex journey as well as relatively nascent and rapidly evolving scholarship on realist syntheses (Rycroft-Malone et al., 2012, Greenhalgh et al., 2017a, Jagosh et al., 2014, Wong et al., 2024), we offer reflections on three study limitations and strengths. First, we did not go through systematic phases of screening, data extraction and quality appraisal and engaged with these in a more iterative and organic manner. While being a potential methodological limitation, such an approach allowed us to draw upon wider body of knowledge in a flexible and adaptive mode without being limited by methodological constraints of the reviewed sources. Our overall learning from this process is that the realist synthesis must be customised to the nature of the questions posed, availability of relevant literature and degree of engagements with empirical data.

Second, given a broad focus of our review question on health systems responsiveness and also reflecting the scarcity of particularly theoretical literature on responsiveness, we spent much more time than planned on identifying relevant theoretical framing of responsiveness. This inevitably means less iterations within the research team on theory-informed programme theories, though the slower pace of working during the COVID-19 pandemic somewhat compensated for that. In contrast with some realist studies, our theory of health systems responsiveness is informed by multiple substantive theories rather than just one theoretical foundation. On reflection, our experience echoes the argument by Jagosh et al. that “…the degree of heterogeneity of the evidence base…[may] determine whether theory can drive the development of review protocols from the outset, or will follow only after an intense period of data immersion” (Jagosh et al., 2014)

Last, we aimed to develop a middle-range theory of health systems responsiveness. At various points in the process, the research team immersed themselves in advancing individual parts of the overall theory – for example, issues of stigma around MMH and integration of MMH services in Vietnam (Thi et al., 2024, Trang et al., 2024a). These somewhat delayed our theorisation of responsiveness as a construct, but these deep dives into contextual and systemic issues ultimately helped better theorisation at middle-range level (Greenhalgh et al., 2017a, Greenhalgh et al., 2017b, Greenhalgh et al., 2017c).

## Conclusions

In this realist synthesis, we bridge the current knowledge gap in health systems responsiveness by deepening the understanding of mechanisms of health systems responsiveness and articulating a programme theory of how responsiveness works. Health systems responsiveness is a socially constructed phenomenon built on the premise of people having agency to ensure and demand social accountability and equity. Different domains of health systems responsiveness overlap, inter-related and comprise: underlying values, potential processes and available resources and context; health systems responsiveness is fundamentally about capacity of people to engage with their health systems and capacity of systems to respond to people’s expectations. Our middle-range theory of health systems responsiveness emphasises the importance of favourable social and organisational context in triggering sense of agency, literacy and empowerment to contribute to enhanced capacity of both the people to engage with their health systems and ability of the health systems to respond to people’s expectations. Future research is needed to apply and test the proposed theory of how health systems responsiveness works in different contexts and health areas.

## Funding sources

The research reported in this paper received funding from the Joint Health Systems Research Initiative comprising Medical Research Council (10.13039/501100000265MRC), Foreign, Commonwealth & Development Office (FCDO) and Wellcome Trust (grant ref: MR/T023481/2). The views are of the authors only and do not necessarily represent those of the funders

## CRediT authorship contribution statement

**Gyimah Leveana:** Writing – review & editing, Investigation, Formal analysis. **Thi Le Minh:** Writing – review & editing, Investigation, Formal analysis. **Trang Do Thi Hanh:** Writing – review & editing, Investigation, Formal analysis. **Kane Sumit:** Writing – review & editing, Investigation, Funding acquisition, Formal analysis, Conceptualization. **Mirzoev Tolib:** Writing – review & editing, Writing – original draft, Methodology, Funding acquisition, Formal analysis, Conceptualization. **Lakin Kimberly:** Writing – review & editing, Investigation, Formal analysis. **Agyepong Irene Akua:** Writing – review & editing, Methodology, Funding acquisition, Conceptualization. **Manzano Ana:** Writing – review & editing, Writing – original draft, Investigation, Funding acquisition, Conceptualization. **Yevoo Linda Lucy:** Writing – review & editing, Methodology, Investigation, Formal analysis. **Ha Bui Thi Thu:** Writing – review & editing, Methodology, Funding acquisition, Conceptualization. **Danso-Appiah Anthony:** Writing – review & editing, Investigation, Funding acquisition, Formal analysis. **Awini Elizabeth:** Writing – review & editing, Investigation, Formal analysis.

## Declaration of Competing Interest

All authors declare no competing interests
