# Supplementary figures and images for: Theoretical foundations and mechanisms of health systems responsiveness: a realist synthesis
Source: SSM Health Syst. 2025 Jun;4:100061. doi: 10.1016/j.ssmhs.2025.100061 (PMC12062191; doi:10.1016/j.ssmhs.2025.100061)

Supplementary file 1: visualisation of emerging programme theories, June 2021


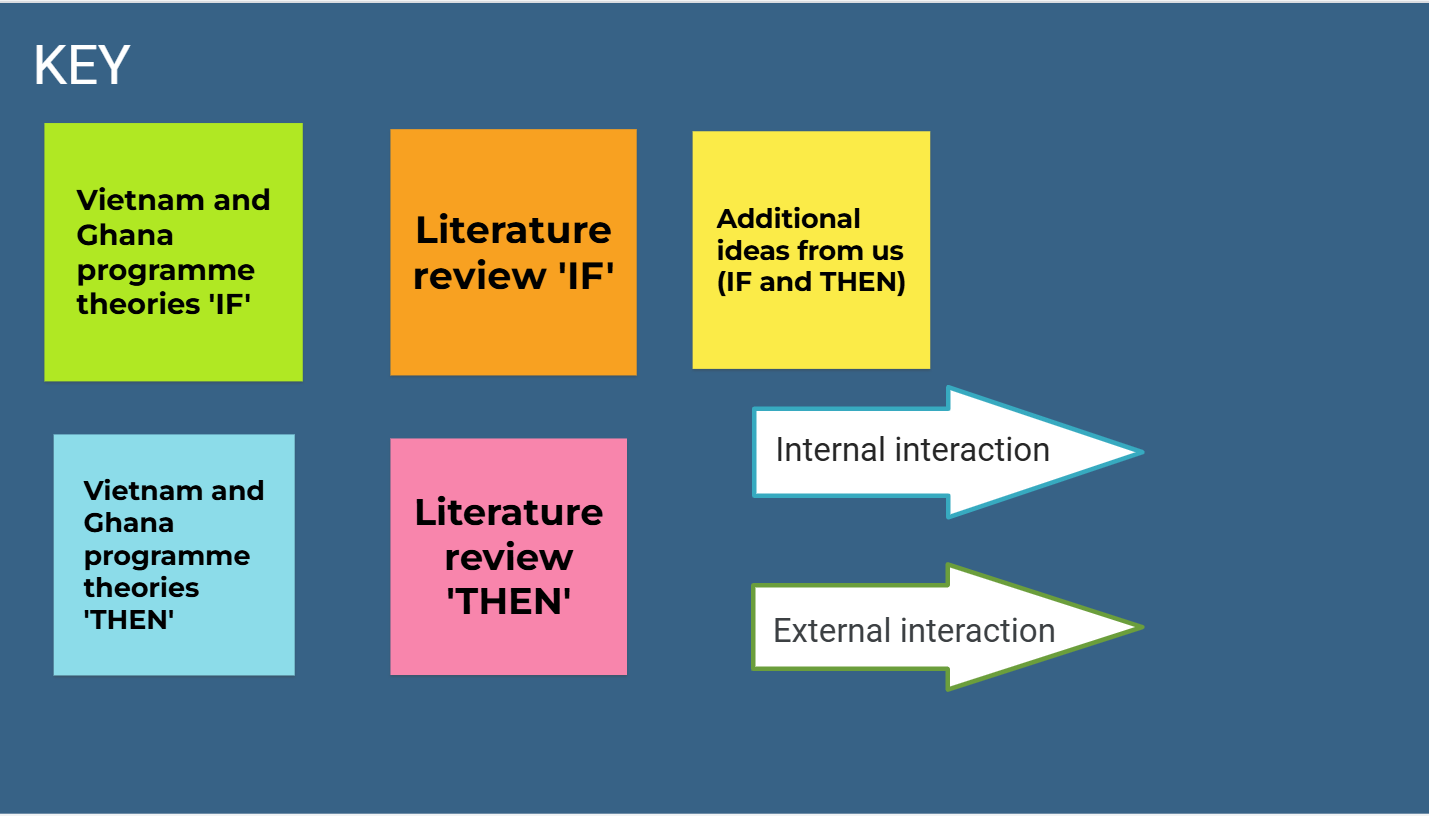


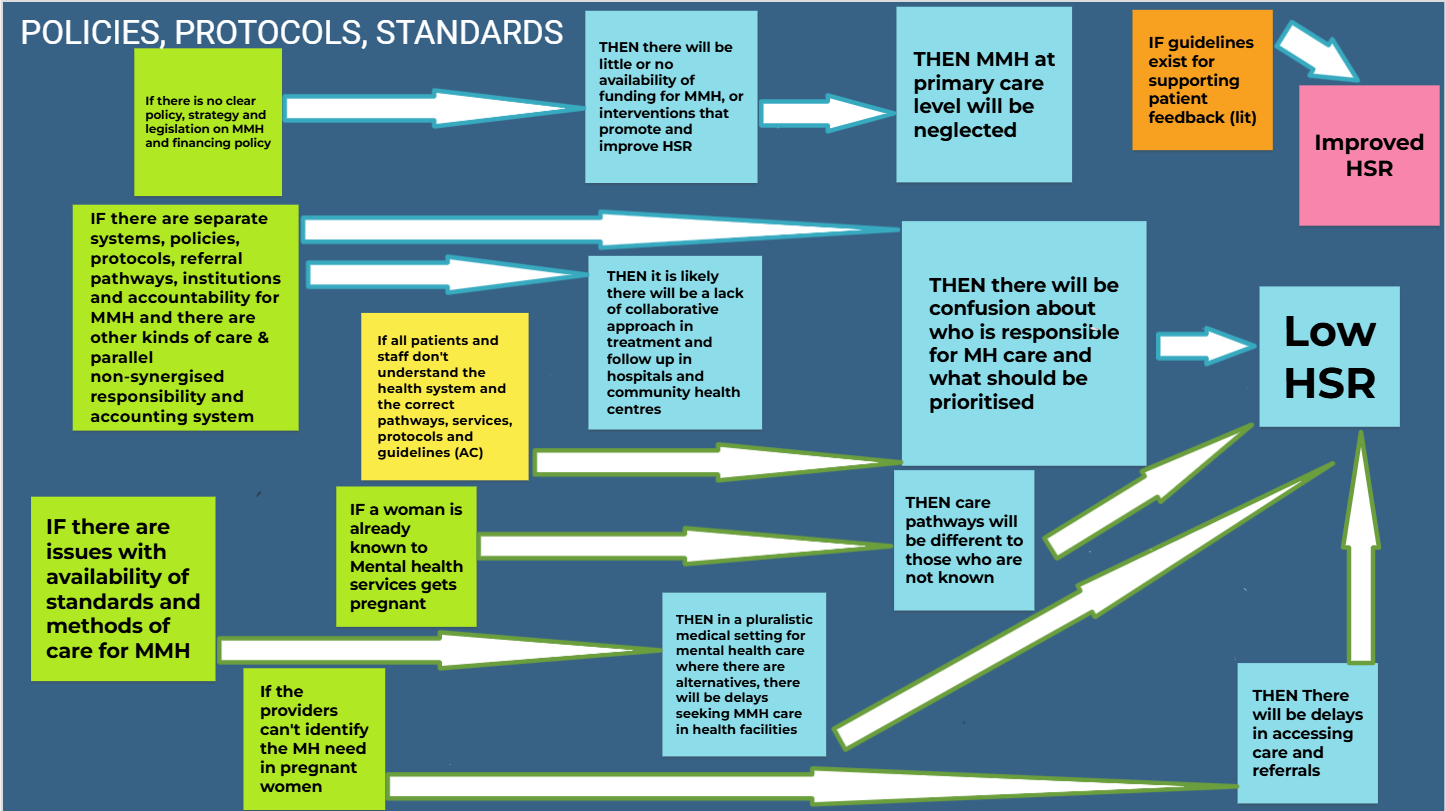


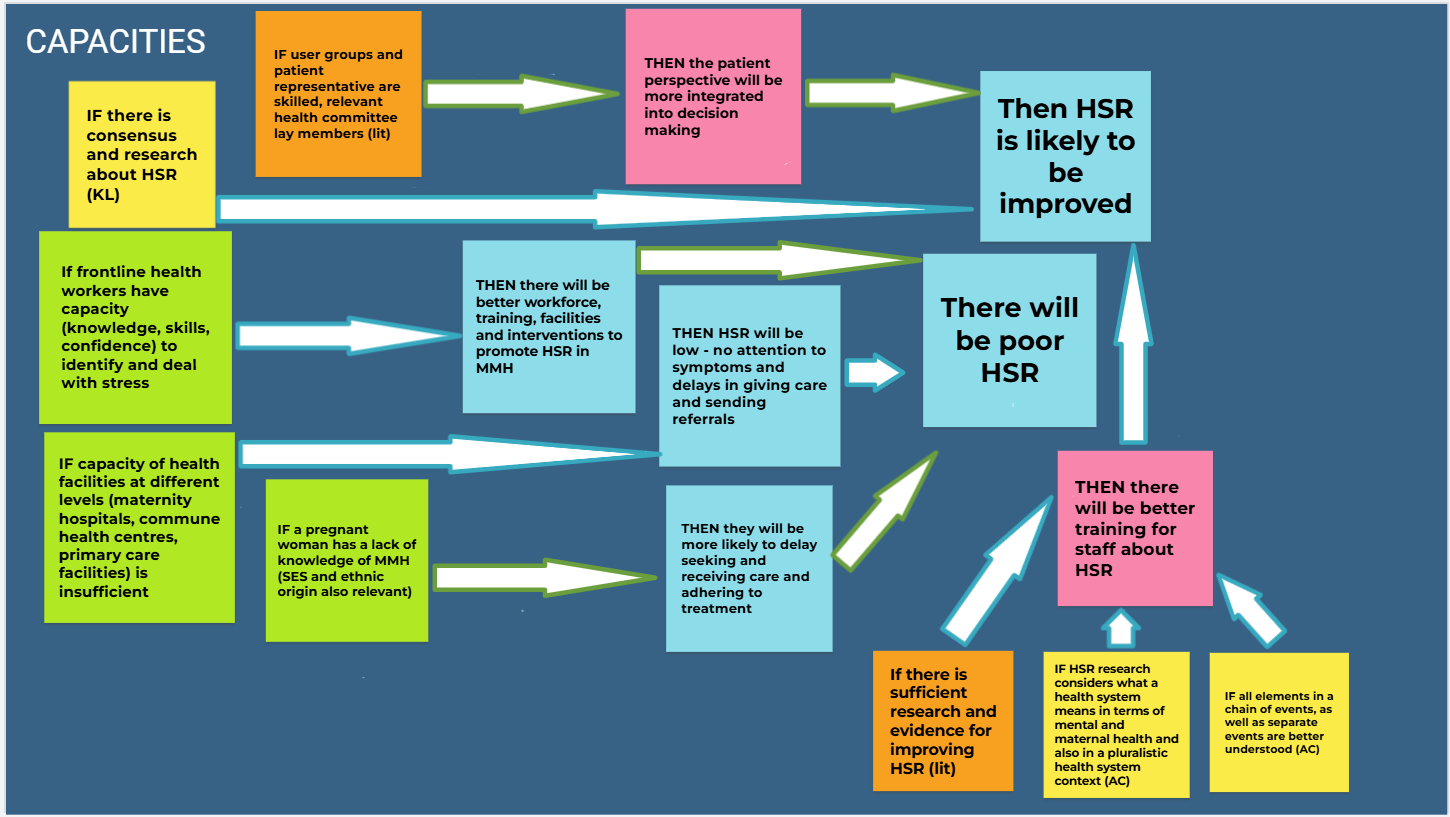


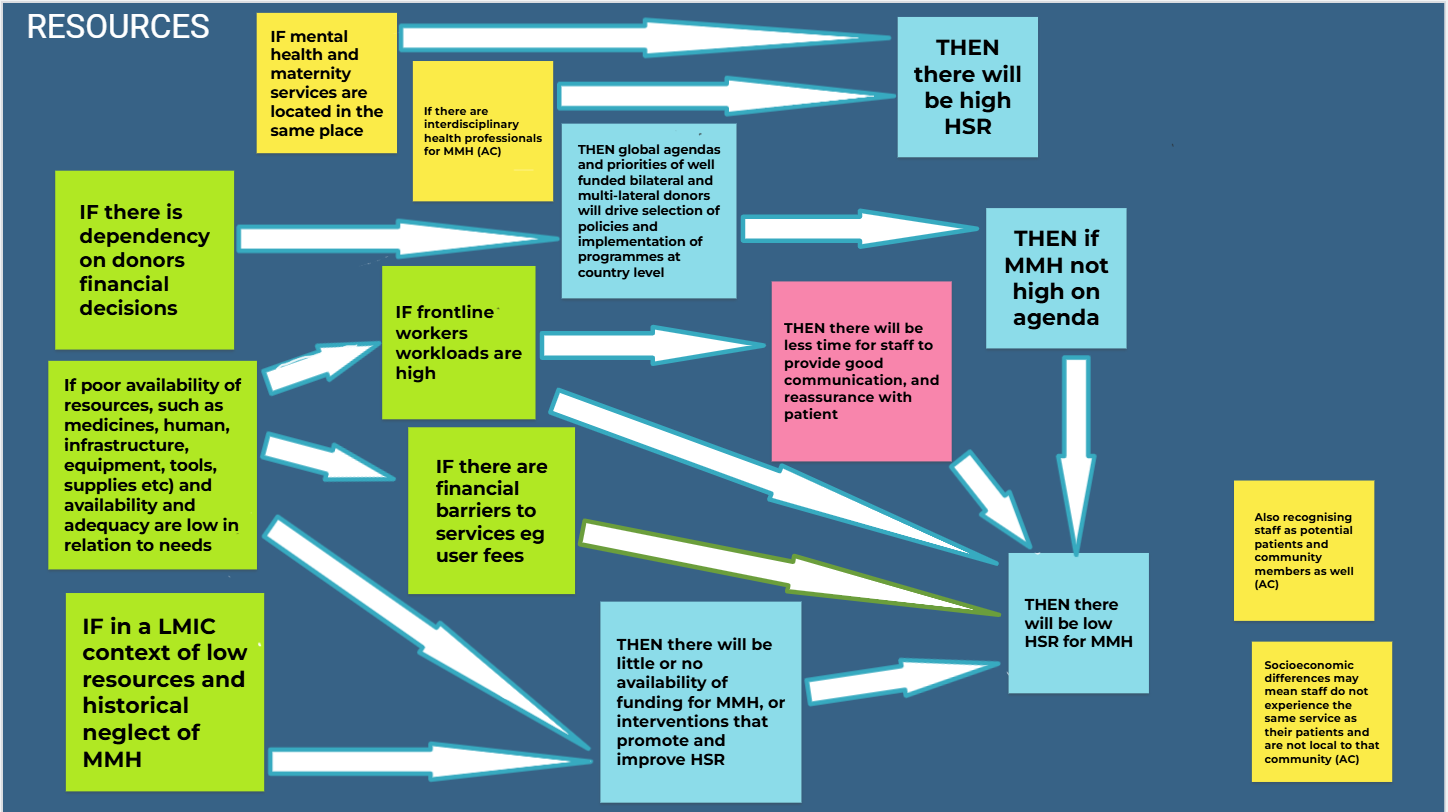


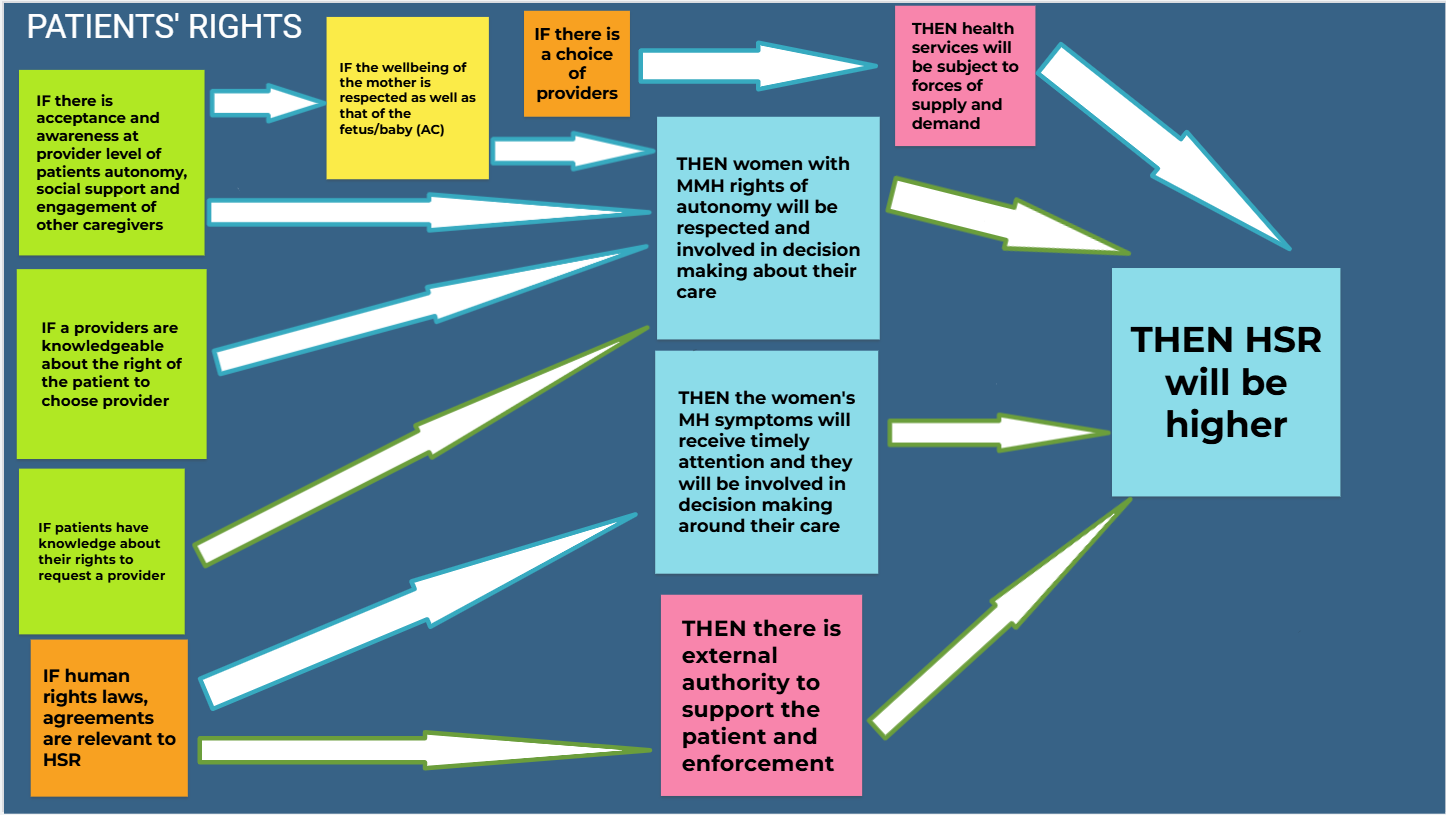


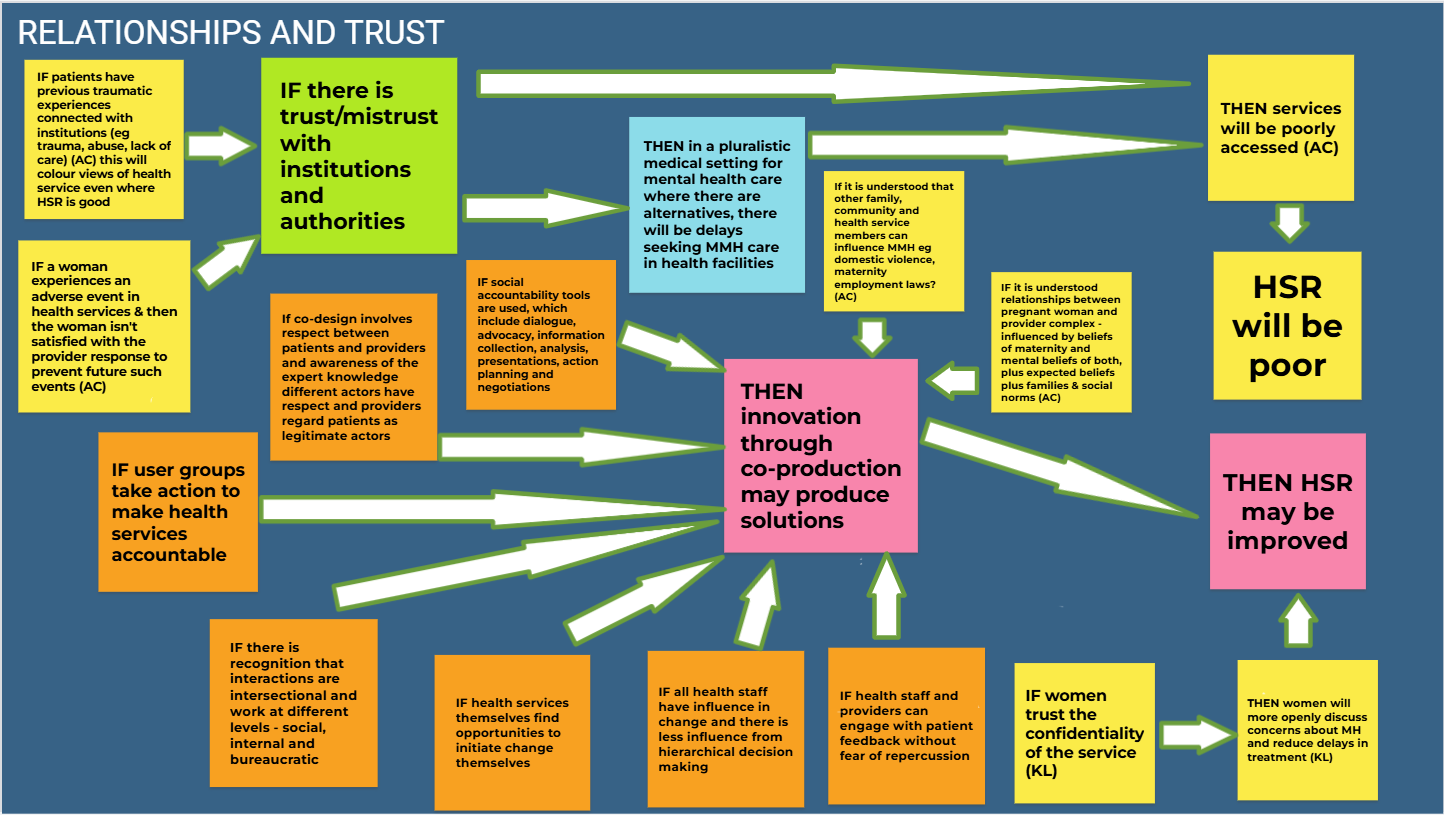


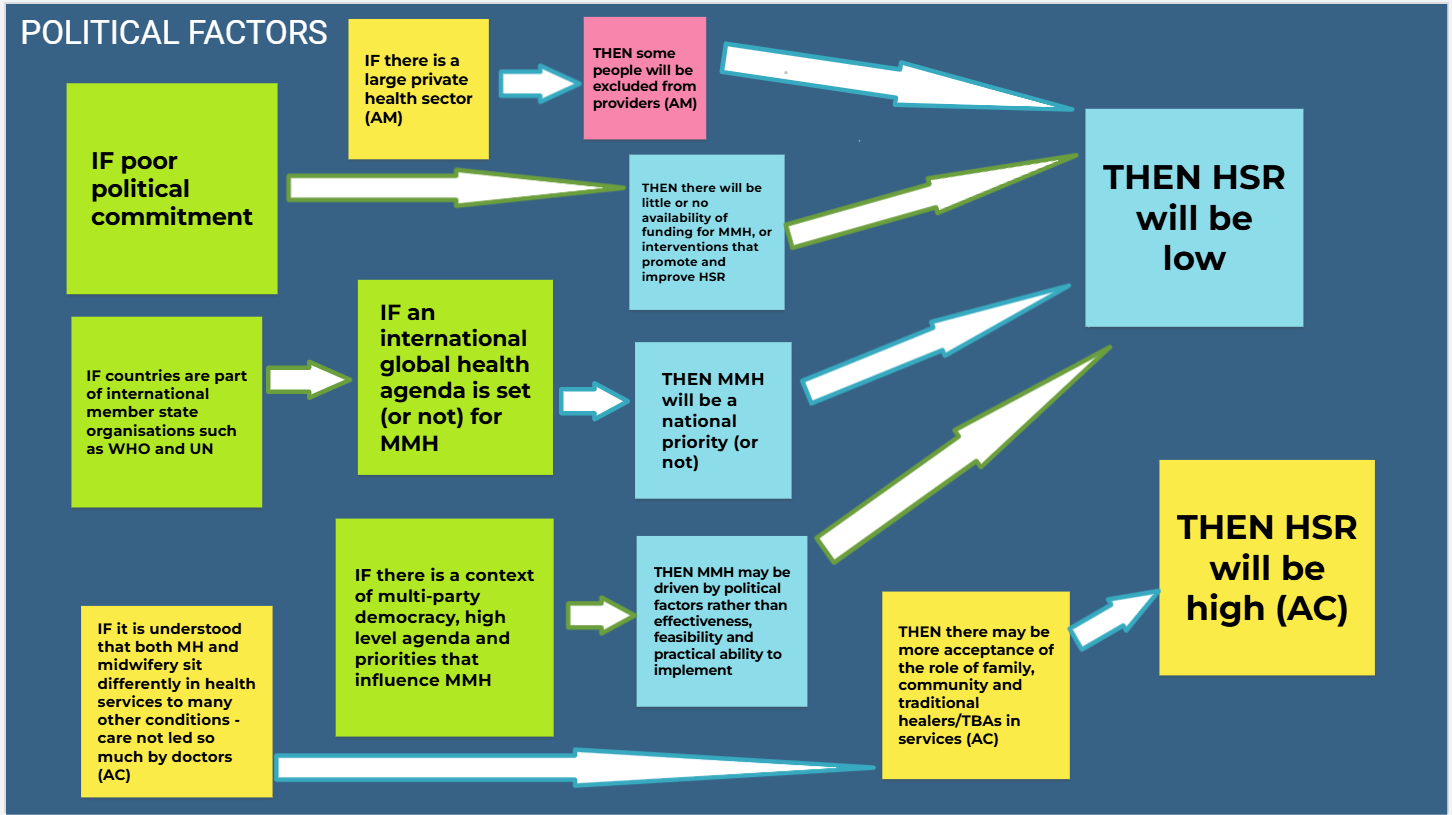


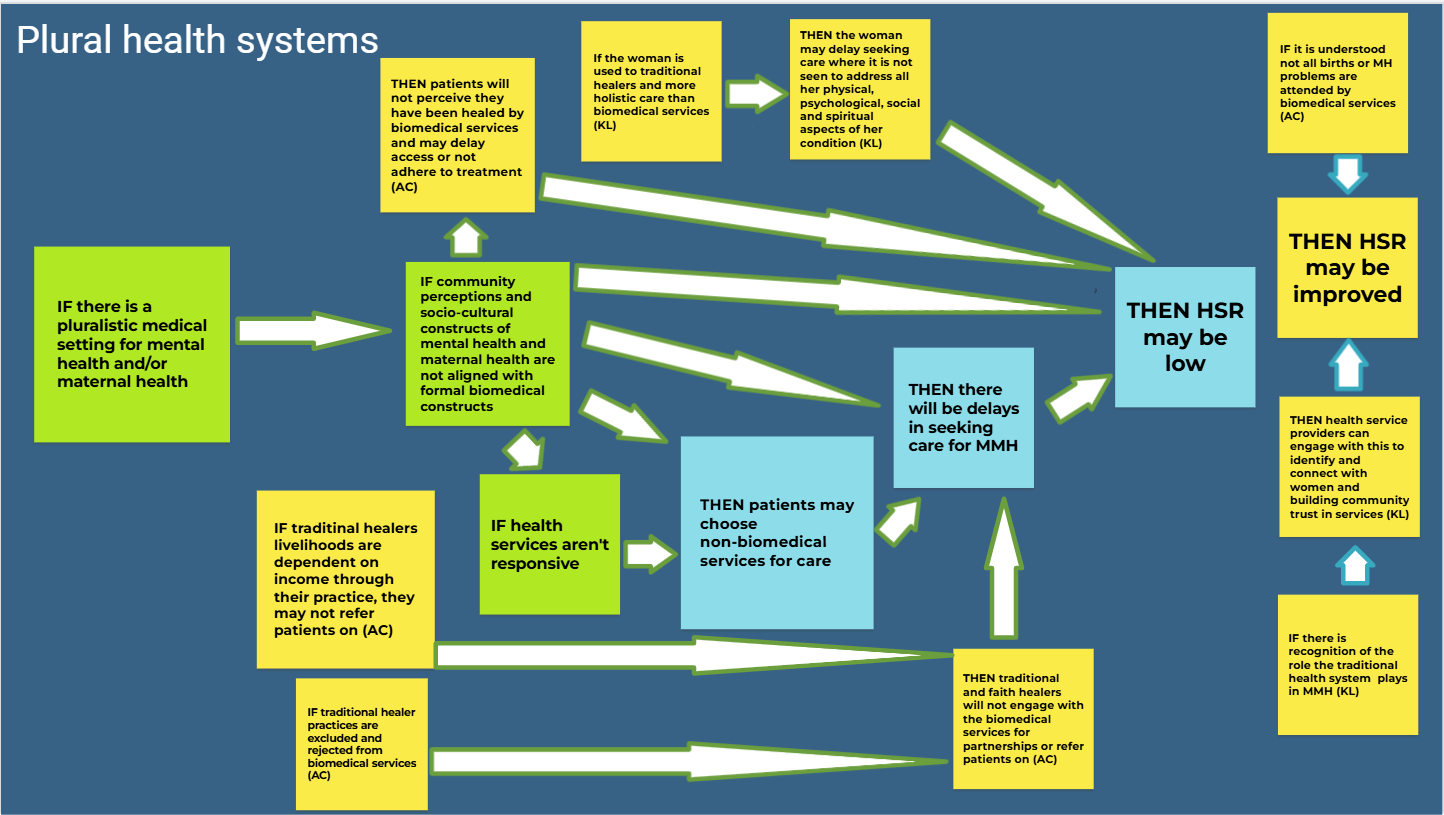


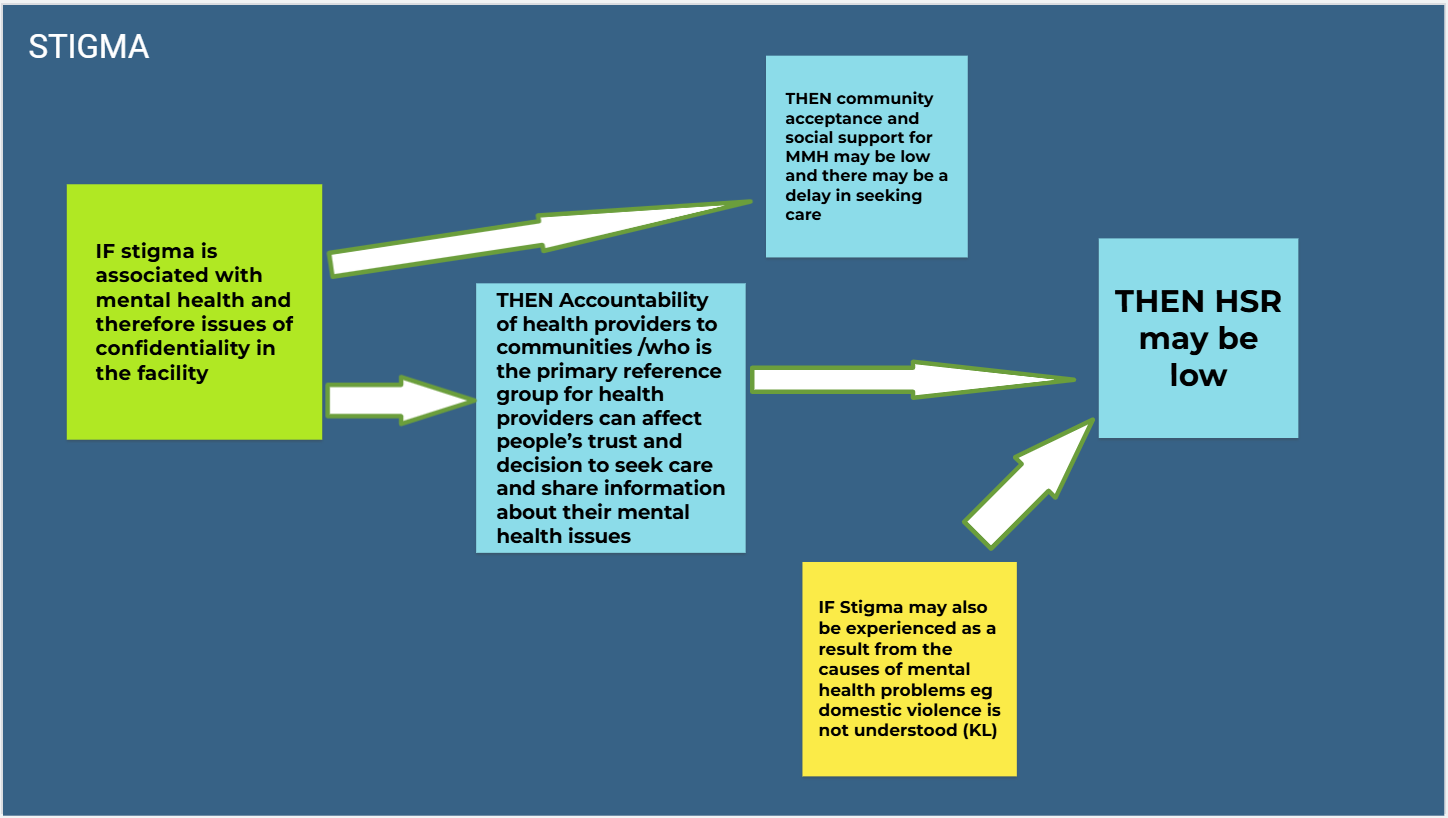


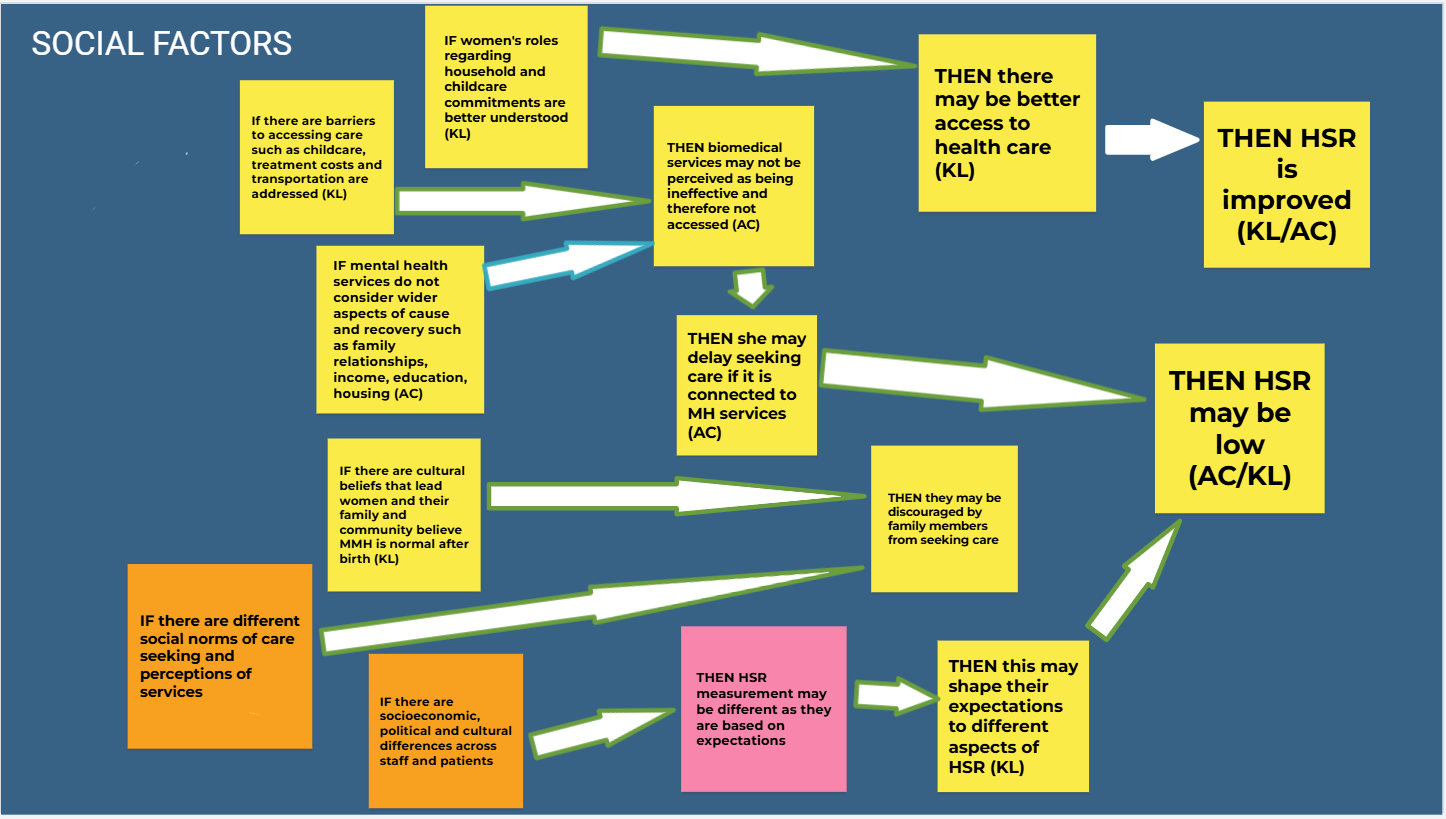


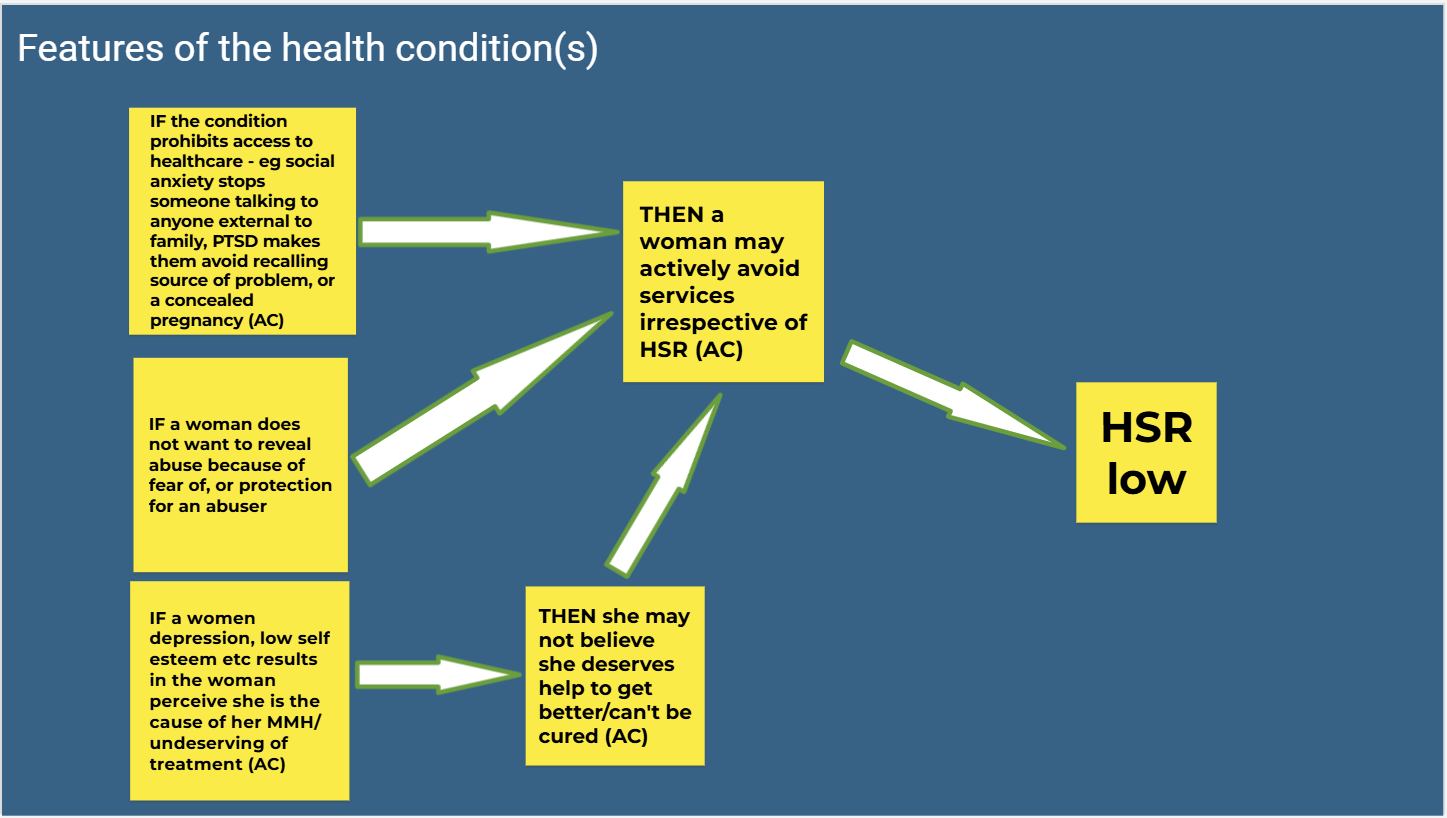

Supplement: Supplementary file 1 — Supplementary material [file mmc1.docx]
